# Residual Volume and Total Lung Capacity at Diagnosis Predict Overall Survival in Non‐Small Cell Lung Cancer Patients

**DOI:** 10.1002/cam4.70962

**Published:** 2025-05-15

**Authors:** Ting Zhai, Yi Li, Robert Brown, Michael Lanuti, Justin F. Gainor, David C. Christiani

**Affiliations:** ^1^ Department of Environmental Health Harvard T.H. Chan School of Public Health Boston Massachusetts USA; ^2^ Department of Biostatistics University of Michigan School of Public Health Ann Arbor Michigan USA; ^3^ Pulmonary and Critical Care Unit, Department of Medicine Massachusetts General Hospital and Harvard Medical School Boston Massachusetts USA; ^4^ Division of Thoracic Surgery, Department of Surgery Massachusetts General Hospital and Harvard Medical School Boston Massachusetts USA; ^5^ Massachusetts General Hospital Cancer Center and Department of Hematology & Oncology Massachusetts General Hospital and Harvard Medical School Boston Massachusetts USA

**Keywords:** non‐small‐cell lung carcinoma, pulmonary function tests, residual volume, survival, total lung capacity

## Abstract

**Background:**

Residual volume (RV) / total lung capacity (TLC) ratio has been found to better predict functional impairments than spirometry and is associated with mortality in chronic obstructive pulmonary disease; however, it is rarely studied in lung cancer. Our previous work established spirometry as a prognostic factor for lung cancer, and we aimed to further investigate the prognostic value of TLC and RV in lung cancer patients.

**Methods:**

We identified newly diagnosed non‐small cell lung cancer (NSCLC) patients who underwent static lung function tests prior to any cancer therapy between 1992 and 2020 in a longitudinal cohort of lung cancer patients: the Boston Lung Cancer Study. Cox proportional‐hazards model was used to estimate the association between each lung volume test with overall survival.

**Results:**

Among 2348 NSCLC patients, 57.2% were diagnosed at stage I and 63.8% underwent surgery, with 1352 deaths observed over a median survival of 66.9 months. Higher RV, RV%, and lower TLC, TLC% were associated with worse overall survival marginally; RV/TLC was associated with overall survival as a quantitative trait, with one standard deviation (11.24%) increase in RV/TLC associated with 19.2% higher risk of mortality (HR = 1.192 [95% CI: 1.114, 1.277]) after covariate adjustment. Statistically significant interactions were found between RV/TLC and spirometry, and higher mortality risks were found with higher RV/TLC in patients across spirometry status and cancer stages.

**Conclusion:**

NSCLC patients with higher RV/TLC ratios at diagnosis had worse overall survival, even when spirometry was within the predicted range. These findings suggest that lung volume measurements provide prognostic information beyond standard spirometry, supporting the need for further mechanistic and interventional studies to determine their clinical utility.

## Graphical abstract

Lung volume as a part of pulmonary function test is often underrepresented in prognostic lung cancer research. In this longitudinal lung cancer patient cohort, higher residual volume to total lung capacity (RV/TLC) ratio upon diagnosis was associated with worse overall survival regardless of spirometry results or cancer stage. The findings provide first‐line evidence for the prognostic value of lung volume measurements and lay the groundwork for future studies to clarify their role in lung cancer prognosis.

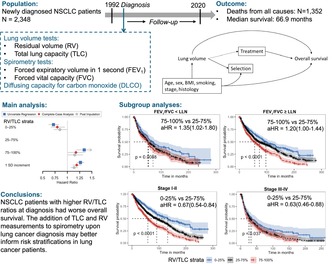

**Abbreviations**
AIS: adenocarcinoma in situ
BLCS: Boston Lung Cancer Study
BMI: body mass index
CI: confidence interval
COPD: chronic obstructive pulmonary disease
DLCO: diffusing capacity for carbon monoxide
FEV_1_
: forced expiratory volume in 1 seconds
FVC: forced vital capacity
HR: hazard ratio
NSCLC: non‐small cell lung cancer
OS: overall survival
PFT: pulmonary function test
RV: residual volume
TLC: total lung capacity

## 1 Introduction

Lung cancer is the leading cause of cancer deaths worldwide, and non‐small cell lung cancer (NSCLC) accounts for approximately 80% of lung cancer [1]. 2‐year survival rates of NSCLC increased from 34% to 42% from 2009 through 2016, likely due to improvements in targeted cancer therapies [2]. Several prognostic factors of lung cancer have been established, including age, sex, smoking, and disease stage [3]. While molecular testing has become increasingly important in guiding personalized treatment, it typically requires invasive biopsy procedures, which might not always be feasible due to clinical constraints, patient condition, or logistical challenges. The identification of phenotypic prognostic factors, which can be readily assessed at the time of diagnosis, remains valuable in complementing molecular markers in cancer prognosis studies. Incorporating phenotypic factors as an adjunct to definitive pathology can refine risk stratification further and optimize therapeutic strategies for patients with NSCLC, thereby potentially contributing to further improvements in survival outcomes.

Pulmonary function correlates with age, and poor pulmonary function is a strong risk factor for all‐cause mortality in the general population [4, 5, 6]. The evaluation of pulmonary function is widely applied in lung disease diagnosis and management, and often consists of spirometry (e.g., forced expiratory volume in 1 s [FEV_1_], forced vital capacity [FVC]), lung volumes (e.g., residual volume [RV], total lung capacity [TLC]) and lung diffusion capacity (e.g., diffusing capacity for carbon monoxide [DLCO]) [7]. For instance, spirometry is commonly used to diagnose and classify disease severity in chronic obstructive pulmonary disease (COPD). “Static” lung volume measures, or simply “volumes” (e.g., RV, the volume of gas remaining in the lungs after a maximal voluntary exhalation; and TLC, the volume of gas in the lungs after a maximal voluntary inhalation) have been reported to better correlate with patient symptoms and impairment of functional capabilities than forced expiratory maneuvers (i.e., spirometry) [8]. RV and the RV/TLC ratio are often used to evaluate pulmonary hyperinflation as COPD progresses [9], and a high RV/TLC ratio has been identified as a risk factor for mortality in patients with COPD [10, 11, 12].

Given the prognostic value of RV and RV/TLC ratio in COPD, and that COPD itself has been found to be an important prognostic factor of lung cancer [13, 14], we evaluated the independent role of lung volumes in predicting survival outcomes in lung cancer patients. To date, most prognostic studies of lung cancer have focused on spirometry measurements [15, 16], with different histological types and at various stages. However, most studies to date are limited by small sample sizes [17, 18, 19, 20, 21, 22, 23]. This study examines the role of TLC and RV in the prognosis of lung cancer in the Boston Lung Cancer Study (BLCS), a large‐scale, lung cancer patient cohort established in 1992. To our knowledge, this is the first study to investigate the relationship between RV and RV/TLC ratio with overall survival in a lung cancer case cohort setting.

## 2 Materials & Methods

### 2.1 Study Design and Participants

**FIGURE 1:**
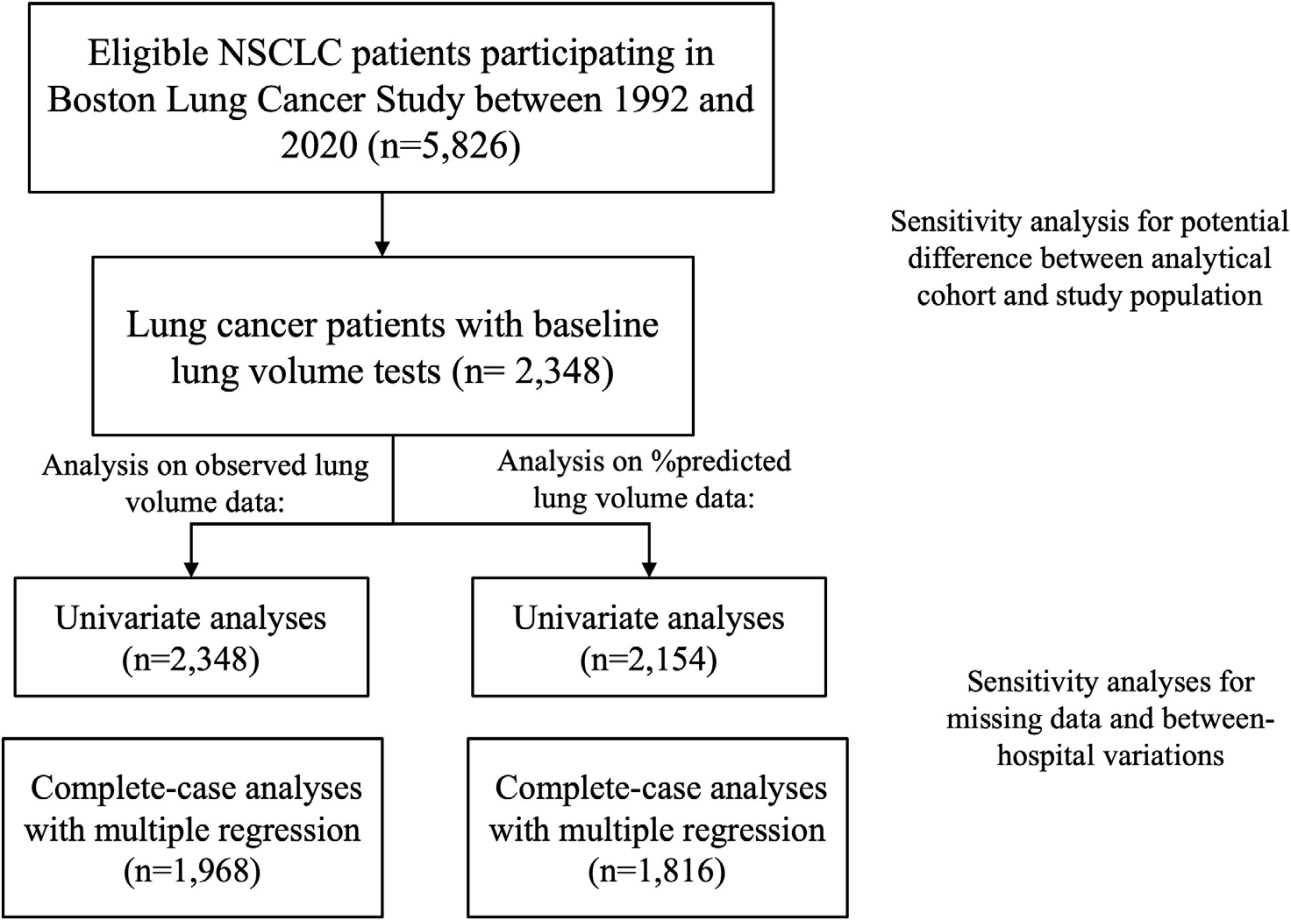
Inclusion for the study population and analytical cohort.

BLCS is a longitudinal, ongoing cancer epidemiology cohort of lung cancer patients established in 1992 at Massachusetts General Hospital (MGH, Boston, MA, USA) [24]. The study enrolls patients newly diagnosed with histologically confirmed lung cancer at the time of recruitment, with no restrictions on stage or treatment type. All patients were 18 years or older at enrollment, and the majority were white (95.3%). Demographic information, including age, sex, body mass index (BMI), and smoking status and intensity, was collected at enrollment by trained staff using a standardized questionnaire. Clinical information, including tumor histology, clinical stage, and initial cancer treatment types, was retrieved retrospectively from electronic medical records. Between 1992 and 2020, among 5940 patients with NSCLC, 5826 patients received follow‐up, and 2348 patients had lung volume and TLC tests performed before initiating lung cancer treatment and were included in our analytical cohort (Figure 1). This study was approved by the institutional review board of MGH (Partners Human Research Committee, Protocol No. 1999P004935/MGH), and informed consent was collected from each study participant or their surrogates.

### 2.2 Pulmonary Function Measurement

TLC and RV test data were obtained from pulmonary function test (PFT) reports or medical records in BLCS, which were entered upon patient enrollment into the electronic medical record system. The majority of PFTs were performed on‐site at MGH during lung cancer diagnosis, following the standards of the American Thoracic Society (ATS)/ European Respiratory Society (ERS). Lung volume tests were primarily measured using body plethysmography or helium dilution techniques [25]. Quality control of collected PFT values was performed by trained staff during data extraction and independently by two pulmonary physicians (RB and DC) post‐data extraction. Data collection and processing were conducted blind to patients' survival status. We excluded PFTs that were performed after the initiation of lung cancer treatment, as treatments could alter pulmonary function.

RV was calculated by subtracting slow vital capacity (SVC) from TLC. The predicted value and lower limit of normal (LLN) of each PFT were calculated through the online calculator of the Global Lung Function Initiative (GLI) (http://gli‐calculator.ersnet.org/index.html) [26]. Tests for survival analysis included RV and percent predicted RV (RV%), TLC and percent predicted TLC (TLC%), RV/TLC ratio, and percent predicted RV/TLC (RV/TLC%). Analysis on percent predicted data was restricted to patients with age < 80 years based on the capability of GLI prediction for lung volumes [26]. Additionally, where available, we collected and calculated diffusing capacity for carbon monoxide (DLCO) and percent predicted DLCO (DLCO%) as our secondary variables of interest. Spirometry, including FEV_1_, percent predicted FEV_1_ (FEV_1_%), FVC, percent predicted FVC (FVC%), and FEV_1_/FVC ratio, was also collected and calculated.

### 2.3 Survival Data Collection

Our primary outcome was overall survival, i.e., the time lag in months between the date of pathological diagnosis of lung cancer and the date of death (event) or the last known date alive (censor), whichever came first. Data were collected from the following sources as previously described [24]: MGH patient and outpatient records, MGH tumor registry, social security death index, primary physician's office, and patient or family contact. As BLCS is an ongoing longitudinal cohort, there was no fixed cut‐off date for follow‐up. Patients who were alive at their last recorded contact were censored at that time, following standard survival analysis procedures. These patients remain under observation unless they withdraw from the study or become untraceable, resulting in a loss to follow‐up rate of 1.9%.

### 2.4 Statistical Analysis

Cox proportional‐hazards (PH) models were used to estimate hazard ratios (HRs) and 95% confidence intervals (CIs) for lung volume with overall survival, modeling time from diagnosis to death (event) or censoring (alive at last follow‐up). To illustrate the shape of the relationship between each lung volume and overall survival, we first fitted penalized smoothing splines and visualized the HRs in response to the changes in each lung volume. This approach also served as our tool to examine the nonlinear associations, given the continuous nature of the lung volume values. In the main analyses, to reduce the impact of outlier data, we categorized values into three percentile groups (1%–25%, 25%–75%, and 75%–100%), and used the 25%–75% group as the reference group. We also modeled each measure on a continuous scale by examining one standard deviation (SD) increments of TLC, RV, and RV/TLC.

Multiple regression models were adjusted for known prognostic factors of lung cancer or potential confounders, including age, sex, BMI, smoking status (never vs. ever, current), NSCLC histological subtypes (adenocarcinoma vs. adenocarcinoma in situ [AIS], squamous cell, large cell, and unspecified), and clinical stages (I vs. II, III, and IV). These patient characteristics were correlated with the tests and believed to be confounders for the associations between RV, TLC, RV/TLC, and overall survival (Figure S1). Because all lung volume tests were performed before any cancer treatment, treatment was hypothesized to mediate the effects of lung volumes and other covariates on survival. To allow patients receiving different treatments to have distinct baseline hazards and to better isolate the association of lung volumes with overall survival, the Cox models were further stratified on lung cancer treatments (surgery only vs. surgery and chemo/radiation, and chemo/radiation only).

The PH assumption of the Cox model was evaluated for each variable by comparing its interaction with follow‐up time, and violations were addressed by including their time interaction terms in each model. The deviance residuals were plotted to evaluate the goodness of fit for the Cox models. Model performance was evaluated by Harrell's C‐index [27].

Complete‐case analyses were conducted in the main analysis as there was no evidence for informative missingness, with a large sample size and a complete case rate of 71.8%. Missing values were imputed based on the mean, median, or mode of observed data [28], and the results based on imputed values were compared to those from complete‐case analyses. We also explored the interactions between RV, TLC, and RV/TLC with spirometry by centering on the person with the median value of each test as the reference, and modeling one SD increment of each test in the Cox model. Further analysis was conducted by stratification on study subjects based on their spirometry status (within the normal range: FEV_1_/FVC equal or above the LLN of GLI reference values, and with abnormal spirometry: FEV_1_/FVC below the LLN) and cancer stage (stage I‐II and stages III‐IV) to evaluate the prognostic values of RV/TLC among patients classified on well‐accepted prognostic factors. Kaplan–Meier curves were compared across the RV/TLC groups within these strata, and survival differences were assessed using log‐rank tests.

To assess potential selection bias in our analytical cohort, we compared demographic and clinical characteristics as well as overall survival between NSCLC patients with and without available lung volume tests in the entire BLCS cohort. Because patient characteristics contributed to test availability (Figure S1), we further evaluated potential selection bias after covariate adjustment in survival analysis. For potential heterogeneity in pulmonary function tests conducted within and outside MGH, we indexed in‐hospital and out‐hospital lung volume tests to assess the effect of between‐hospital variation by examining its interaction with each lung volume test variable.

All *P*‐values were two‐sided, and *p* < 0.05 was considered statistically significant. Statistical analyses were conducted using SAS (version 9.4; SAS Institute Inc., Cary, NC, USA) and R (version 4.0.2; R Foundation for Statistical Computing).

## 3 Results

### 3.1 Patient Characteristics

Table 1 describes the demographic and clinical information of 2348 NSCLC patients in the analytic cohort (mean [SD] age: 67.49 [9.85] years, 49.74% male patients). The majority of patients were diagnosed at stage I (57.2%) and received surgery as their primary treatment (63.8%). A total of 1362 deaths were observed with a median survival of 66.9 months (95% confidence interval [CI]: 60.4–73.0 months). The 1‐, 5‐, and 10‐year survival rates were 86.2%, 52.5%, and 34.6%, respectively, and the median follow‐up length was 40.5 months (range, 0.07–281.54 months). From the univariate analyses, age, sex, BMI, smoking status, pack‐years, NSCLC histological subtypes, clinical cancer stages, and post‐diagnosis cancer treatments were all significantly associated with overall survival in lung cancer patients (Table 1).

**TABLE 1:** Characteristics of NSCLC patients and associations with overall survival.

Patient Characteristics	Distribution	HR (95% CI)
Age (years)	67.49 (9.85)	1.026 (1.020 1.032)
Male sex	1147 (49.74)	1.230 (1.104 1.369)
Height (meters)	1.67 (0.11)	1.403 (0.825 2.385)
BMI (kg/m^2^)	26.68 (5.39)	0.982 (0.971 0.993)
Smoking status		
Never smoker	260 (11.35)	—
Previous smoker	1387 (60.54)	1.618 (1.323 1.979)
Current smoker	644 (28.11)	1.940 (1.573 2.393)
Pack‐years	37.52 (16.60–59.00)	1.007 (1.006 1.009)
NSCLC histology		
Adenocarcinoma	1397 (59.50)	—
AIS	168 (7.16)	0.777 (0.637 0.949)
Squamous cell	529 (22.53)	1.688 (1.489 1.914)
Large cell	98 (4.17)	1.832 (1.465 2.291)
NSCLC‐unspecified	156 (6.64)	1.443 (1.171 1.779)
Stage		
I	1328 (57.19)	—
II	442 (19.04)	2.442 (2.130 2.800)
III	443 (19.08)	2.417 (2.111 2.768)
IV	109 (4.69)	5.367 (4.303 6.695)
Lung cancer treatment		
Surgery only	1437 (63.81)	—
Surgery + Chemo/Radiation	442 (19.63)	1.340 (1.166 1.540)
Chemo/Radiation	373 (16.56)	4.103 (3.585 4.697)

*Note:* Data are presented as mean (SD) for age, BMI, and median (IQR) for pack‐years, No. (%) for categorical variables. They were summary statistics of the observed data (with missing rate ranging from 1.07% to 19.64%, respectively). Abbreviations: AIS, adenocarcinoma in situ; BMI, body mass index; NSCLC, non‐small cell lung cancer.

TLC, RV, and diffusion capacity test results are summarized in Table 2. RV/TLC had a mean of 47.7% (SD: 11.2%) and was well‐correlated with FEV_1_ (*Pearson* correlation coefficient [*r*] = −0.70, *p* < 0.001), FEV_1_% (*r* = −0.62, *p* < 0.001), FVC (*r* = −0.65, *p* < 0.001), and FVC% (*r* = −0.58, *p* < 0.001) (Figure S2).

**TABLE 2:** Distribution of lung volumes and diffusing capacity in the analytical ohort.

PFT	*N*	Mean (SD)	Cut‐off values		
			0%–25%	25%–75%	75%–100%
RV (Liters)	2348	2.81 (1.04)	0.23–2.12	2.12–3.31	3.31–9.88
RV %	2154	128.52 (49.12)	11.46–104.10	104.10–161.10	161.10–497.28
TLC (Liters)	2348	5.84 (1.37)	1.96–4.87	4.87–6.65	6.65–15.09
TLC %	2154	100.85 (20.10)	36.49–88.11	88.11–112.40	112.40–274.83
RV/TLC	2341	47.71 (11.24)	8.7–39.8	39.8–54.7	54.7–87.2
RV/TLC %	2154	135.79 (32.83)	31.69–112.84	112.84–155.30	155.30–298.31
DLCO (mL/min/mmHg)	2158	16.59 (6.14)	1.30–12.30	12.30–20.50	20.53–44.51
DLCO %	2123	75.69 (25.05)	5.59–59.03	59.03–92.13	92.13–188.23

Abbreviations: DLCO%, percent predicted diffusing capacity for carbon monoxide; DLCO, diffusing capacity for carbon monoxide; PFT, pulmonary function test; RV, residual volume; SD, standard deviation; TLC, total lung capacity.

### 3.2 RV, TLC Predicts Overall Survival in NSCLC

**FIGURE 2:**
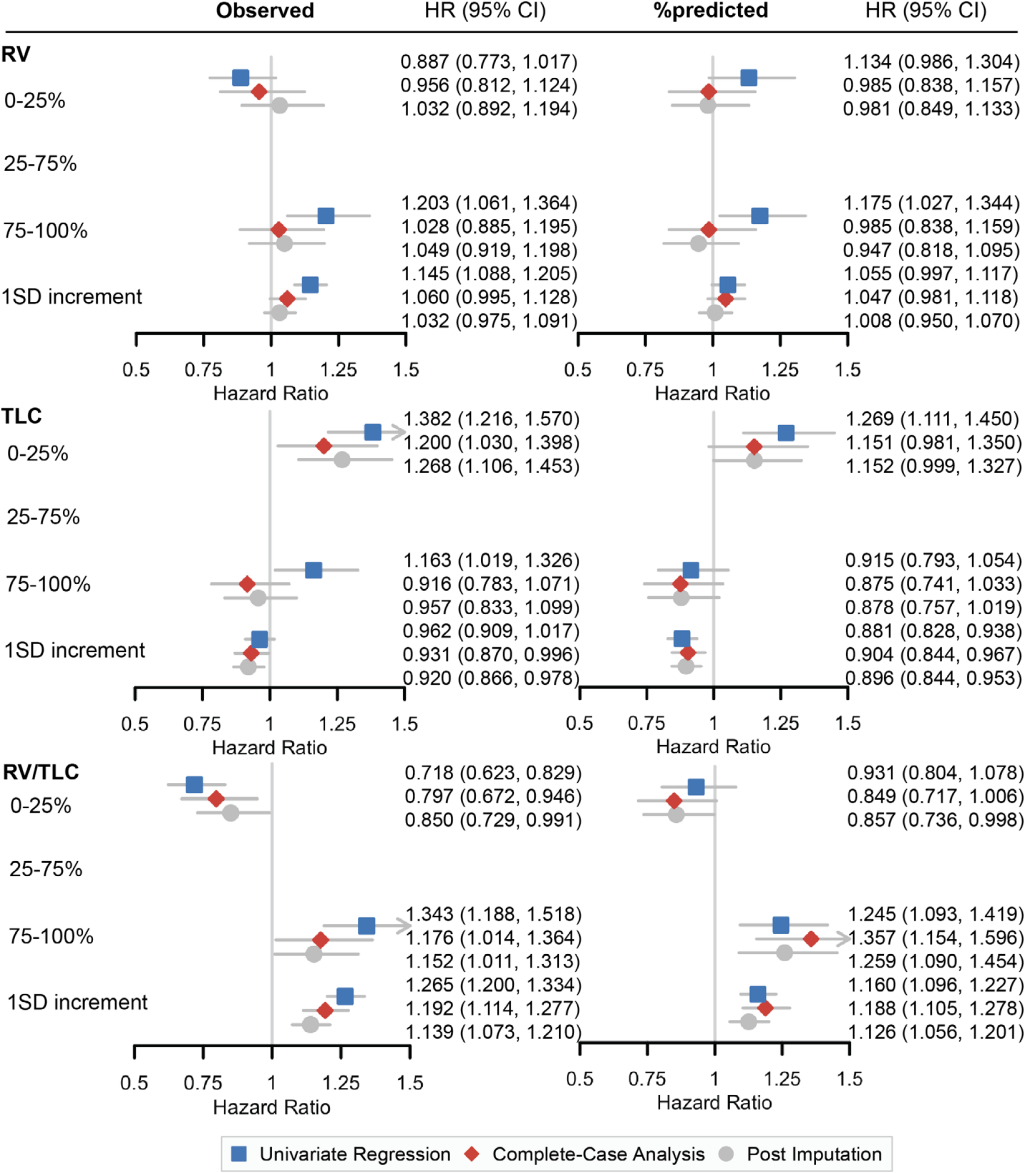
Associations between lung volumes and risk of death in NSCLC patients. Associations with observed RV, TLC RV/TLC are on the left, and associations with RV%, TLC%, and RV/TLC% are on the right. Hazard ratios are shown in comparison to the reference 25%–75% category for the 0%–25% and 75%–100% categories, as well as in response to a 1 standard deviation increment of each lung volume test. Complete‐case analysis and post‐imputation analysis were ed. for age, sex, BMI, smoking, NSCLC histological subtypes, clinical stages, lung cancer treatments, and their time‐varying effects. Arrows indicate intervals out of range (0.5–1.5). Abbreviations: HR = hazards ratio; CI = confidence interval; SD = standard deviation; RV = residual volume; TLC = total lung capacity.

The crude and adjusted HRs of death by each static lung volume test are summarized in Figure 2. In the univariate models, compared to patients with average lung volume (having RV and TLC in the 25–75 percentile), patients with good lung volume (in the 0–25 percentile for RV, RV%, RV/TLC, and RV/TLC%) had lower mortality rates, and patients with resting pulmonary hyperinflation (in the 75–100 percentile for RV, RV%, RV/TLC, and RV/TLC%) had higher mortality rates. The associations of RV/TLC and RV/TLC% with survival remained significant after controlling for age, sex, BMI, smoking status, NSCLC histological subtypes, clinical stages, lung cancer treatments, and their time‐varying effects. Similarly, patients with poor TLC and TLC% (in the 0–25 percentile) had higher mortality rates, while those in the 75–100 percentile had lower mortality rates.

Among all measures, RV/TLC showed the strongest dose‐dependent association with overall survival, where higher values were associated with worse outcomes. In unadjusted models, patients in the 75–100th percentile for RV/TLC had a 34.3% higher mortality risk compared to those in the 25–75th percentile (HR = 1.343, 95% CI: [1.188, 1.518]), whereas those in the 0–25th percentile had a 28.2% lower mortality risk (HR = 0.718, 95% CI: [0.623, 0.829]). After covariate adjustment, patients in the 75–100th percentile had a 17.6% higher risk of death compared to those in the 25–75th percentile (adjusted HR = 1.176, 95% CI: [1.014, 1.364]), whereas patients in the 0–25th percentile had a 20.3% lower risk of death (adjusted HR = 0.797, 95% CI: [0.672, 0.946]). In contrast, after covariate adjustment, the associations of other lung function measures (RV, RV%, TLC, TLC%) with overall survival were no longer statistically significant (Figures 2 and S3).

Consistent with the survival analysis based on percentile groups, a 1 SD increment of RV, RV%, RV/TLC, and RV/TLC% was associated with higher mortality rates, and a 1 SD increment of TLC and TLC% was associated with lower mortality rates (Figure 2). The strongest association was observed for RV/TLC, where a 1 SD increase was associated with a 19.2% increase in mortality risk after covariate adjustment (adjusted HR = 1.192, 95% CI: [1.114, 1.277]).

### 3.3 RV/TLC Interacts With FEV_1_ % and FVC% on Mortality Risks

One SD increment of FEV_1_, FEV_1_%, FVC, and FVC% was all significantly associated with lower mortality rates in univariate and multiple regression models (Figure S4). Given the correlations between RV/TLC and spirometry tests and the strong association between RV/TLC and overall survival, we further explored how RV/TLC interacts with spirometry tests in predicting mortality risks in NSCLC patients.

Significant interactions between RV/TLC and FEV_1_% (adjusted interaction HR = 1.087, 95% CI: [1.018, 1.160]) as well as FVC% (adjusted interaction HR = 1.096, 95% CI: [1.030, 1.168]) were found, suggesting a higher mortality risk with increased RV/TLC when the spirometry test remained consistent. Interestingly, better spirometry tests remained significant predictors of improved overall survival in the interaction models, where the mortality rates were compared per one SD increment of each spirometry test among patients with median values of RV/TLC (Figure S4).

### 3.4 Prognostic Values of RV/TLC Are Independent of Spirometry and Cancer Stage

**FIGURE 3:**
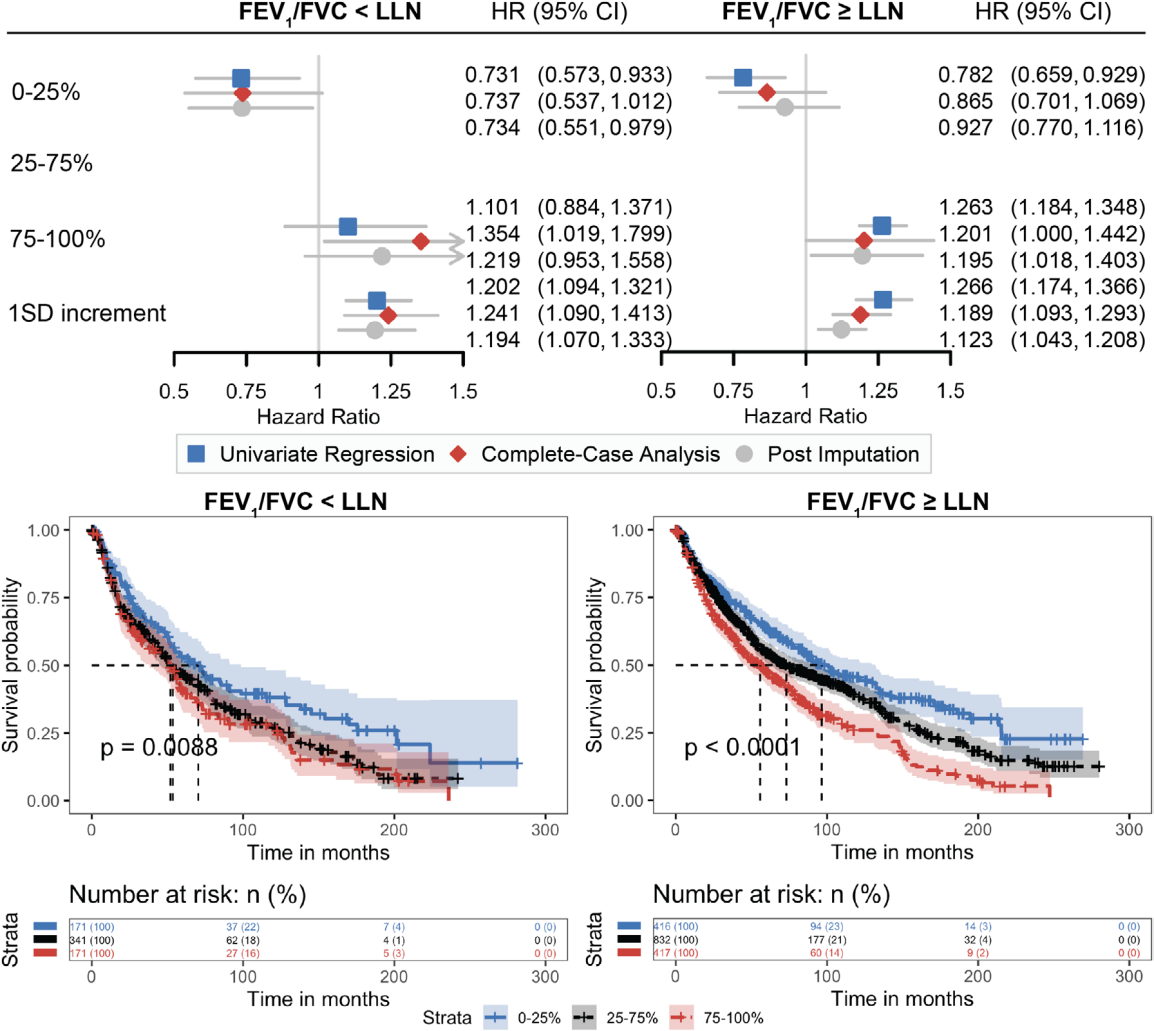
RV/TLC and RV/TLC% predict risk of deaths independent of spirometry status. Hazard ratios are shown in comparison to the reference 25%–75% category for the 0%–25% and 75%–100% categories, as well as in response to a 1 standard deviation increment of each lung volume test. Complete‐case analysis and post‐imputation analysis were adjusted for age, sex, BMI, smoking, NSCLC histological subtypes, clinical stages, lung cancer treatments, and their time‐varying effects. Arrows indicate intervals out of range (0.5–1.5). CI, confidence interval; FEV_1_, forced expiratory volume in 1 s; FVC, forced vital capacity; HR, hazards ratio; LLN, lower limit of normal; RV, residual volume; SD, standard deviation; TLC, total lung capacity.

Given the significant interactions found between spirometry and RV/TLC, we explored further the prognostic values of RV/TLC in patients with abnormal spirometry (FEV_1_/FVC<LLN of GLI reference values) and in patients within the range of normal spirometry (FEV_1_/FVC≥LLN) separately, especially among the latter who might be misclassified as at lower risks of death if relying solely on spirometry (Figure 3). Among patients with poor spirometry results, patients having less air trapping defined by RV/TLC (in the 0–25 percentile) had lower mortality rates and better overall survival (median survival = 70.4, 95% CI: [52.3, 98.6] months). And among patients within the range of predicted normal spirometry (FEV_1_/FVC≥LLN), those with hyperinflation at RV defined by RV/TLC (in the 75–100 percentile) had higher mortality rates and worse overall survival (median survival = 55.8, 95% CI: [44.8, 67.0] months). Moreover, one SD increment of RV/TLC was significantly associated with worse overall survival among both patients with both good and poor spirometry (adjusted HR =1.189 [95% CI: 1.093, 1.293] and 1.241 [95% CI: 1.090, 1.413], respectively). These findings presented the consistent values of RV/TLC in predicting the risk of death regardless of spirometry test results.

**FIGURE 4:**
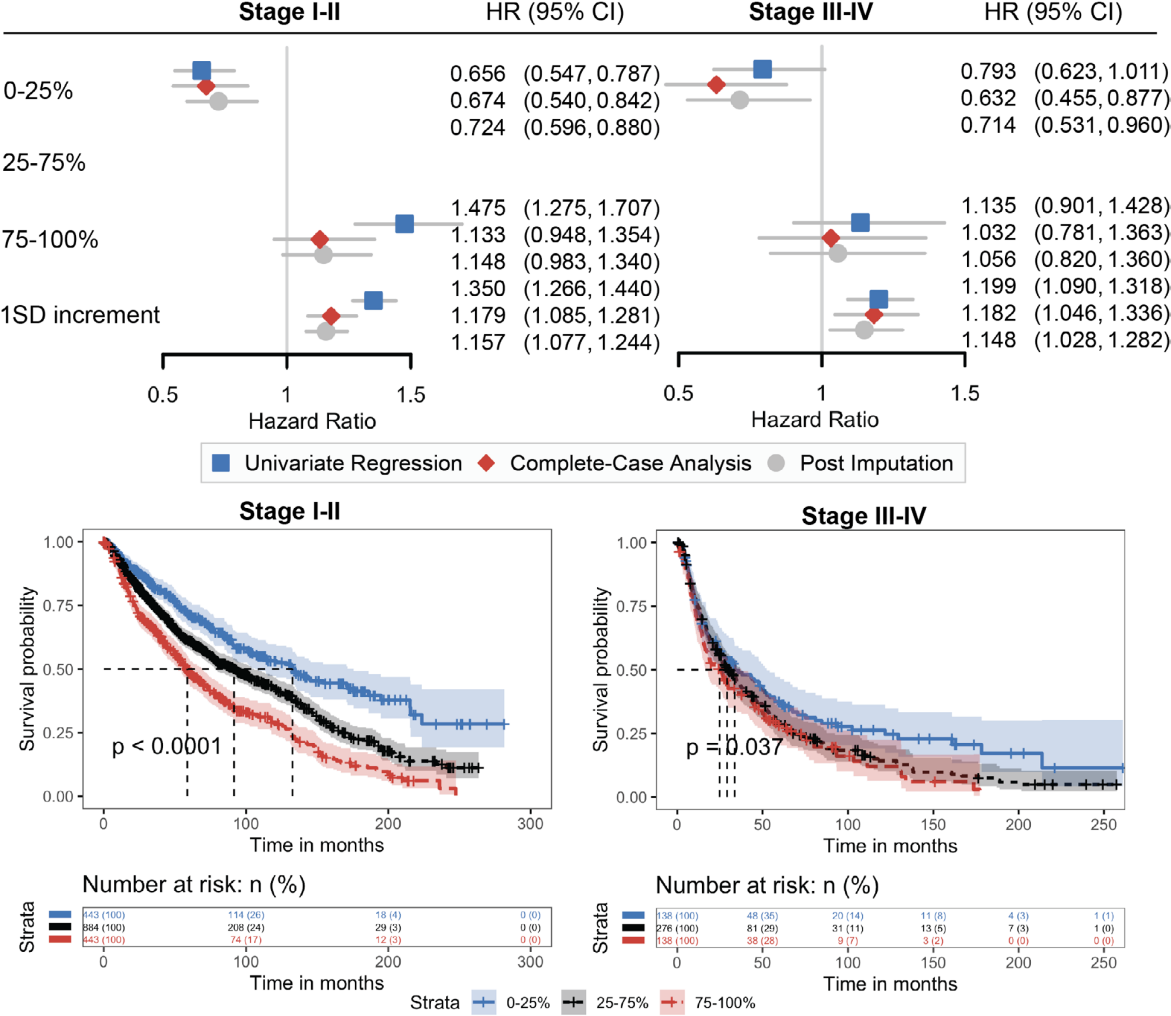
Risk of death associated with RV/TLC in early‐ and late‐stage NSCLC patients. Hazard ratios are shown in comparison to the reference 25%–75% category for the 0%–25% and 75%–100% categories, as well as in response to a 1 standard deviation increment of each lung volume test. Complete‐case analysis and post‐imputation analysis were adjusted for age, sex, BMI, smoking, NSCLC histological subtypes, clinical stages, lung cancer treatments, and their time‐varying effects. Arrows indicate intervals out of range (0.5–1.5). CI, confidence interval; FEV_1_, forced expiratory volume in 1 s; FVC, forced vital capacity; HR, hazards ratio; RV, residual volume; SD, standard deviation; TLC, total lung capacity.

Next, we evaluated the prognostic role of RV/TLC in patients with NSCLC diagnosed at early stages and at advanced stages (Figure 4). The median survival was 86.2 months (95% CI: 78.0, 93.6) for stage I‐II patients and 28.1 months (95% CI: 24.9, 34.2) for stage III‐IV patients. Among both early‐stage (log‐rank *p* < 0.0001) and advanced‐stage (log‐rank *p* = 0.037) patients, survival outcomes differed significantly across RV/TLC percentile groups. In stage I‐II patients, those in the 0–25th percentile had the best survival (median = 132.6, 95% CI: [106.2, 175.6] months), whereas those in the 75–100th percentile had the poorest survival (median = 58.7, 95% CI: [52.0, 69.9] months). A similar trend was observed in stage III–IV patients, with a median survival of 33.6 months (95% CI: 24.6, 52.3) in the 0–25th percentile group, compared to 24.9 months (95% CI: 17.2, 37.5) in the 75–100th percentile group. After covariate adjustment, absence of air trapping with RV/TLC in the 0–25 percentile of both groups was associated significantly with lower mortality rates. Specifically, stage I‐II patients in the 0–25th percentile had a 32.6% lower mortality risk (adjusted HR = 0.674, 95% CI: [0.540, 0.842]), while stage III–IV patients in the same group had a 36.8% lower mortality risk (adjusted HR = 0.632, 95% CI: [0.455, 0.877]). One SD increment of RV/TLC was associated with a 17.9% increase in mortality risk for stage I–II patients (adjusted HR = 1.179, 95% CI: [1.085, 1.281]) and an 18.2% increase in mortality risk for stage III–IV patients (adjusted HR = 1.182, 95% CI: [1.046, 1.336]).

### 3.5 Additional Findings

Univariate analyses of both DLCO (HR = 1.709 [95% CI: 1.500, 1.947] and HR = 0.720 [95% CI: 0.619, 0.838], for 0–25 and 75–100 percentiles vs. 25–75 percentile, respectively) and DLCO% (HR = 1.840 [95% CI: 1.617, 2.093] and HR = 0.696 [95% CI: 0.598, 0.811], for 0–25 and 75–100 percentiles vs. 25–75 percentile, respectively) showed a dose‐dependent association with overall survival, while the relationship of DLCO% became less clear after adjusting for covariates in multiple regression.

### 3.6 Sensitivity Analysis

Model diagnostics using the Deviance Residuals suggested adequate goodness‐of‐fit of these multiple regression models (Figure S5). The C‐indexes were similar (range: 0.958–0.961) for all multiple regression models. The imputation‐based results agreed with those of the complete‐case analyses with slightly attenuated associations, which were expected as imputed values are prone to measurement errors and might thus reduce the predictive power of models [29].

Compared with NSCLC patients without reproducible lung function tests in the BLCS cohort, patients in our analytical cohort were older, with higher BMI, more likely to be smokers, less likely to have NSCLC of adenocarcinoma, and more likely to be in earlier stages and receive surgical treatment (Table S1). As a result, overall survival was lower in patients without reproducible tests in the BLCS cohort (median survival [95% CI]: 32.3 [29.9, 35.2] vs. 67.0 [60.4, 73.1] months), but the difference became insignificant after adjusting for covariates used in the multiple regression analyses (HR comparing patients with and without reproducible tests [95% CI] = 0.930 [0.852, 1.015]). Moreover, the source of lung function test records (inside vs. outside of MGH) was not associated with overall survival in the multiple regression models.

## 4 Discussion

In this long‐term, large clinical cohort of patients with NSCLC, we investigated the association of lung volume tests with overall survival. Our findings demonstrated a robust, quantitatively dependent relationship between RV/TLC and overall survival, independent of known prognostic factors such as age, sex, smoking, histological subtype, stage, and treatment. The RV/TLC ratio exhibited considerable variability across the cohort, ranging from 8.7% to 87.2% (mean: 47.7%, SD: 11.2%), with each SD increase associated with a 19.2% higher risk of death from all causes. Moreover, statistically significant interactions were observed between RV/TLC and FEV_1_%, as well as FVC%, in relation to overall survival. Collectively, these results underscore the potential utility of lung volume as an independent prognostic marker in NSCLC, supplementing the prognostic value provided by commonly accepted clinical parameters.

Because lung function tests are routinely performed before surgery [7], our study cohort included a higher proportion of early‐stage patients, with 57.2% diagnosed at stage I, compared to the broader NSCLC population. Advanced‐stage patients who underwent lung volume testing were generally more functionally preserved, contributing to the prolonged survival (median: 28.1 months) observed in these patients. Nonetheless, 552 participants (23.8%) had stage III‐IV cancer, enabling us to evaluate RV/TLC in a subgroup often underrepresented in prognosis studies. Even here, RV/TLC remained significantly associated with higher mortality (18.2% increased risk per one SD). When comparing RV/TLC percentiles, we observed a 73.9‐month survival benefit among the lower 25% versus the upper 25% in stage I‐II patients (median survival: 132.6 vs. 58.7 months), while in stages III‐IV, the difference was 8.7 months (33.6 vs. 24.9 months), underscoring RV/TLC's prognostic value across NSCLC stages.

Our findings concerning the RV/TLC ratio in NSCLC are consistent with previous literature in COPD patient mortality, as a higher RV/TLC ratio is indicative of lung hyperinflation resulting from decreased elasticity and increased airway resistance [9]. Lung hyperinflation has been shown to impact breathing significantly, and lung volume reduction surgeries can improve expiratory muscle function, exercise capacity, and quality of life [30, 31]. Compared to other common airway obstruction measurements (e.g., FEV_1_), RV and TLC correlate more directly with the functional impairments that might influence symptoms and physical activities [8]. Categorizing COPD patients based on level of dyspnea has been demonstrated to be a better predictor of survival in COPD patients than FEV_1_, which is often used as a measure of disease severity [10]. FEV_1_ and other spirometry measures have been associated with overall survival in NSCLC patients in a quantitative, dose‐dependent manner in our previous study [13]. Here, we demonstrated the prognostic role of the RV/TLC ratio for overall survival in NSCLC patients. The association between lower DLCO and DLCO% with worse overall survival also agrees with previous findings in lung cancer patients [17, 18, 19].

After observing significant correlations between RV/TLC and spirometry tests, we explored further their interactive roles in predicting overall survival. The model performance in predicting survival, as evaluated by C‐index, did not change after incorporating the interaction terms, but the significant interactions between RV/TLC with FEV_1_% and FVC% suggested that at least part of the association between spirometry and mortality was through its interaction with abnormal RV/TLC. Stratifying on spirometry results by FEV_1_/FVC demonstrated that patients with normal‐range spirometry (FEV_1_/FVC≥LLN) yet exhibiting hyperinflation at RV/TLC experienced even shorter survival compared to those with abnormal spirometry (FEV_1_/FVC<LLN) but less air trapping (median survival: 55.8 vs. 70.4 months). Moreover, higher RV/TLC was associated with higher mortality rates in both groups (18.9% increased risk in normal spirometry vs. 24.1% increased risk in abnormal spirometry). This underscores the potential underestimation of risk when relying solely on spirometry, particularly in patients who initially present within the normal range of spirometry tests. The potential lung hyperinflation at RV, which may not be detected by spirometry but is captured by RV/TLC, could be a significant predictor of poor patient survival.

Our results demonstrate that RV/TLC has independent prognostic value for overall survival in NSCLC patients, even among patients whose spirometry appears normal. However, integrating a new prognostic marker into clinical practice requires a multi‐step process involving validation across diverse populations, mechanistic insights, and demonstration of clinical utility. To fully establish the role of RV/TLC, further research is needed to validate its prognostic value in external cohorts and to investigate the underlying biological mechanisms. Additionally, studies should explore whether interventions to improve pre‐treatment pulmonary function are feasible and beneficial, and clinical trials are needed to determine whether identifying patients with abnormally high RV/TLC can guide treatment adaptations, such as refining surgical candidacy, pre‐surgery pulmonary management, post‐treatment monitoring, or supportive care, to improve survival and quality of life.

We acknowledge several limitations to this study. First, the estimated associations between the RV/TLC ratio and long‐term survival in NSCLC patients could be subject to unmeasured confounding bias. The unmeasured confounders might lead to bias in either direction, but we estimated that the magnitude of confounding should be minimal as we adjusted for measured confounders to the extent possible, including cancer stage, NSCLC histological subtypes, and initial treatments. Second, there may be selection bias with the patients included in the analyses being different from the study population. However, the non‐differential mortality risks between patients being included versus patients being excluded after covariate adjustment in sensitivity analysis suggested that we have accounted for factors contributing to the selection, thus mitigating selection bias through blocking the causal pathway via selection (Figure S1). Last, although we enrolled a substantial number of stage III‐IV patients, our analytical cohort was primarily stage I. We found significant associations between RV/TLC and survival in both early and advanced stages, but caution is warranted when generalizing these results to patient populations with predominantly advanced stages.

We evaluated the independent impacts of RV, TLC, and RV/TLC, as well as DLCO, on overall survival in a large cohort of lung cancer patients. After accounting for age, sex, smoking, tumor histology, cancer stage, and types of treatment, higher RV/TLC ratio and lower DLCO were each independently associated with worse overall survival. Previous studies of lung cancer prognosis with lung function tests typically did not account for various histological types and treatment options for lung cancer because of data limitations. By leveraging a long‐established longitudinal lung cancer cohort with complete follow‐up information, we were able to explore a comprehensive set of prognostic factors, reducing the possibilities of residual confounding and selection biases, confirming the prognostic relevance of RV/TLC across diverse stages of NSCLC and spirometry. Most importantly, lung volume is often overlooked in lung cancer prognostic studies, and our findings point to RV/TLC as a valuable, measurable factor that warrants further mechanistic and interventional studies to fully elucidate its clinical utility.

## Author Contributions

**Ting Zhai:** data curation (lead), formal analysis (lead), methodology (lead), visualization (lead), writing – original draft (lead). **Yi Li:** funding acquisition (lead), methodology (equal), supervision (equal), writing – review and editing (equal). **Robert Brown:** conceptualization (equal), investigation (equal), writing – review and editing (equal). **Michael Lanuti:** resources (equal), writing – review and editing (equal). **Justin F. Gainor:** resources (equal), writing – review and editing (equal). **David C. Christiani:** conceptualization (lead), funding acquisition (lead), investigation (lead), project administration (lead), supervision (lead), writing – review and editing (lead).

## Ethics Statement

This study was approved by the institutional review board of MGH (Partners Human Research Committee, Protocol No. 1999P004935/MGH).

## Consent

Written informed consent was obtained from each study participant or their surrogates.

## Conflicts of Interest

The authors declare no conflicts of interest.

## Supporting information


Data S1.

## Data Availability

The data that supports the study findings are available from the corresponding author upon reasonable request.
