# Supplementary material for: Residual Volume and Total Lung Capacity at Diagnosis Predict Overall Survival in Non‐Small Cell Lung Cancer Patients
Source: Cancer Med. 2025 May 15;14(10):e70962. doi: 10.1002/cam4.70962 (PMC12079642; doi:10.1002/cam4.70962)
Supplement: Supplementary file 1 — Data S1. [file CAM4-14-e70962-s001.docx]

**Supplementary materials**

**Supplementary tables**

**Table S1.** Comparison for demographic and clinical characteristics between NSCLC patients with /without valid lung volume test at diagnosis in the BLCS cohort (Available / Unavailable) and between NSCLC patients with /without in-hospital test in the analytical cohort (In-hospital/ Out-hospital).

**Supplementary figures**

**Figure S1. Directed acyclic graph for relationships between lung volume, overall survival, and patient characteristics.**

**Figure S2. Correlations among pulmonary function tests in the Boston Lung Cancer Study.** The heatmap illustrates Pearson’s correlation strength (gradient color) and multiple testing adjusted significance levels (***P<0.001, **P<0.01, *P<0.05) among the pulmonary function tests. Abbreviations: FEV1 = forced expiratory volume in 1 second; FVC = forced vital capacity; DLCO = diffusing capacity for carbon monoxide; DLCO% = percent predicted diffusing capacity for carbon monoxide; RV = residual volume; TLC = total lung capacity.

**Figure S3. Shape of associations between lung volumes and risk of death in NSCLC patients.** Unadjusted (black dot-dashed lines) and adjusted hazard ratios (red solid line) with 95% confidence intervals (blue dashed lines) were obtained from spline Cox regression model of overall survival with each lung volume test as a continuous predictor. The X axis ranges from the 5^th^ to 95^th^ percentile of each test. Abbreviations: RV = residual volume; TLC = total lung capacity.

**Figure S4. RV/TLC versus spirometry: individual effects and interactions on risk of death in NSCLC patients.** The hazard ratios were reported as relative changes per 1 standard deviation increment in the corresponding covariates. Individual effects were measured without adjusting for another variable of interest or their interactions; interactions were reported as hazard ratios of each term of interest in the interaction model. Adjusted hazard ratio denotes the association accounting for age, sex, BMI, smoking, NSCLC histological subtypes, clinical stages, lung cancer treatments and their time varying effects. Abbreviations: NSCLC = non-small cell lung cancer; FEV_1_ = forced expiratory volume in 1 second; FVC = forced vital capacity; RV = residual volume; TLC = total lung capacity.

**Figure S5. Deviance plots for Cox models in the main analyses.** RV (A), RV% (B), TLC (C), TLC% (D), RV/TLC (E), RV/TLC% (F), DLCO (G), DLCO% (H) were modeled with adjustments for age, sex, BMI, smoking status, pack-years, histology, stage, and stratified on treatment.

| **Table S1.** **Comparison for demographic and clinical characteristics between NSCLC patients with /without valid lung volume test at diagnosis in the BLCS cohort (Available / Unavailable) and between NSCLC patients with /without in-hospital test in the analytical cohort (In-hospital/ Out-hospital).** | | | | |
| --- | --- | --- | --- | --- |
| **Variable** | **Available**  **(*n* = 2,348)** | **Unavailable**  **(*n* = 3,478)** | **In-hospital**  **(*n* = 636)** | **Out-hospital**  **(n = 199)** |
| Age* | 67.49 (9.85) | 65.08 (11.04) | 67.39 (10.00) | 65.54 (11.48) |
| Male sex | 1,147 (49.74) | 1,686 (49.07) | 296 (46.98) | 84 (44.92) |
| Height* | 1.67 (0.11) | 1.69 (0.39) | 1.67 (0.11) | 1.67 (0.10) |
| BMI* | 26.68 (5.39) | 26.12 (5.32) | 26.25 (5.22) | 26.65 (5.09) |
| Smoking status*^ | | | |  |
| Never smoker | 260 (11.35) | 518 (15.25) | 62 (9.87) | 29 (17.37) |
| Previous smoker | 1,387 (60.54) | 1,942 (57.17) | 321 (51.11) | 84 (50.30) |
| Current smoker | 644 (28.11) | 937 (27.58) | 245 (39.01) | 54 (32.34) |
| Pack-years*^ | 37.52 (16.60 - 59.00) | 33.78 (9.60 – 55.43) | 39.64 (20.36 – 60.13) | 27.32 (3.11-45.82) |
| NSCLC histology* | | | |  |
| Adenocarcinoma | 1,397 (59.50) | 2,157 (62.02) | 297 (46.70) | 119 (59.80) |
| AIS | 168 (7.16) | 178 (5.12) | 85 (13.36) | 8 (4.02) |
| Squamous cell | 529 (22.53) | 656 (18.86) | 157 (24.69) | 45 (22.61) |
| Large cell | 98 (4.17) | 153 (4.40) | 23 (3.62) | 5 (2.51) |
| NSCLC-unspecified | 156 (6.64) | 334 (9.60) | 74 (11.64) | 22 (11.06) |
| Stage*^ | | | |  |
| I | 1,328 (57.19) | 1,235 (35.93) | 298 (47.45) | 107 (54.31) |
| II | 442 (19.04) | 939 (27.32) | 135 (21.50) | 39 (19.80) |
| III | 443 (19.08) | 595 (17.31) | 147 (23.41) | 47 (23.86) |
| IV | 109 (4.69) | 668 (19.44) | 48 (7.64) | 4 (2.03) |
| Lung cancer treatments*^ |  |  |  |  |
| Surgery only | 1,437 (63.81) | 1,269 (40.11) | 263 (43.76) | 109 (59.24) |
| Surgery + Chemo/Radiation | 442 (19.63) | 509 (16.09) | 172 (28.62) | 48 (26.09) |
| Chemo/Radiation | 373 (16.56) | 1,386 (43.81) | 166 (27.62) | 27 (14.67) |
| Data are presented as mean (SD) for age, BMI, and median (IQR) for pack-years, No. (%) for categorical variables. They were summary statistics of the observed data (with missing rate ranging from 1.07% to 19.64%, respectively).  *P<0.05 for the comparison between patients with valid lung volume tests and without in the BLCS cohort.  ^P<0.05 for the comparison between patients with lung volume tests performed within MGH and outside from MGH in the analytical cohort.  Abbreviations: BMI = body mass index; AIS = adenocarcinoma in situ; BLCS = Boston Lung Cancer Study; NSCLC = non-small cell lung cancer. | | | | |

**
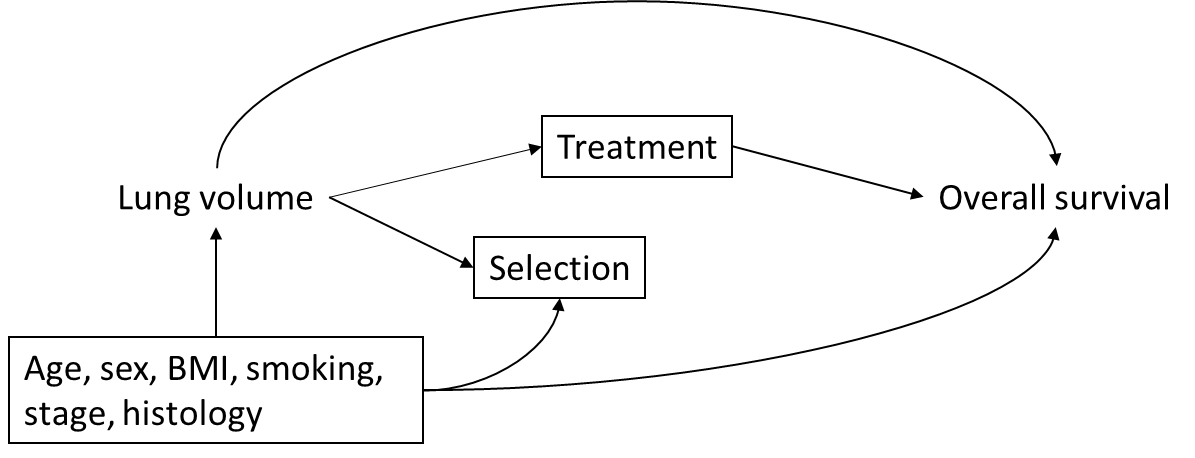
**

**Figure S1. Directed acyclic graph for relationships between lung volume, overall survival, selection, and patient characteristics.**

**
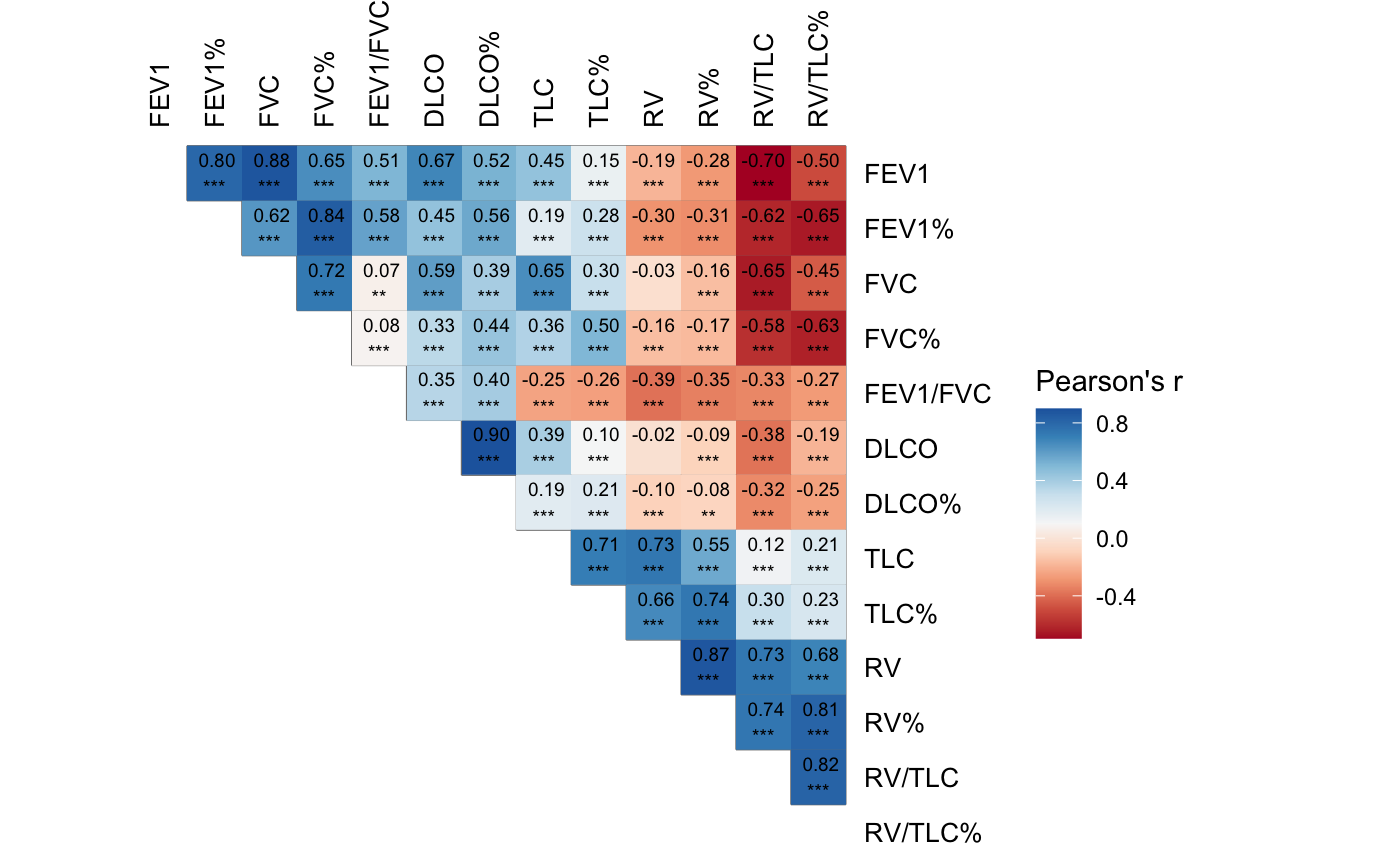
Figure S2. Correlations among pulmonary function tests in the Boston Lung Cancer Study.** The heatmap illustrates Pearson’s correlation strength (gradient color) and multiple testing adjusted significance levels (***P<0.001, **P<0.01, *P<0.05) among the pulmonary function tests. Abbreviations: FEV1 = forced expiratory volume in 1 second; FVC = forced vital capacity; DLCO = diffusing capacity for carbon monoxide; DLCO% = percent predicted diffusing capacity for carbon monoxide; RV = residual volume; TLC = total lung capacity.

| 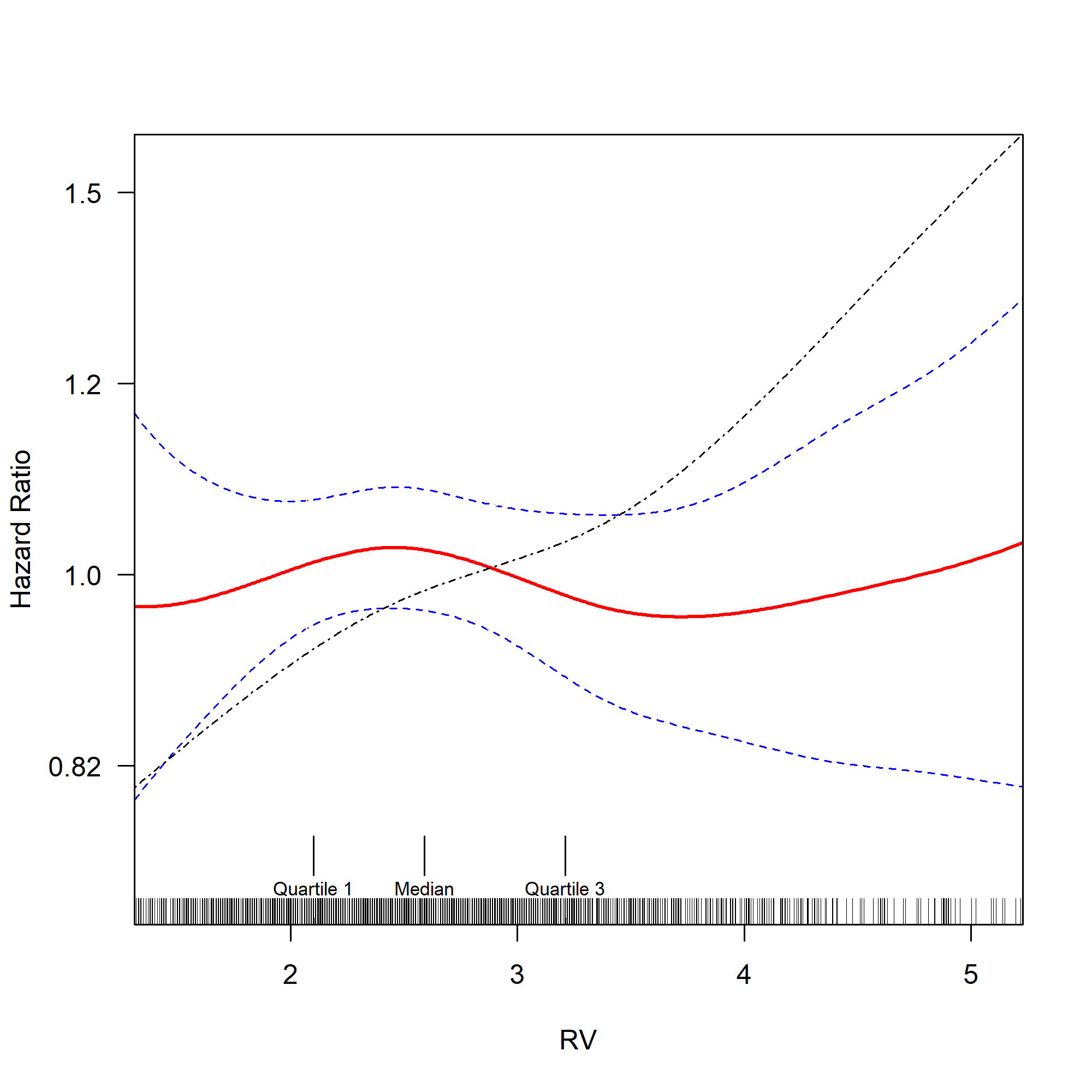 | 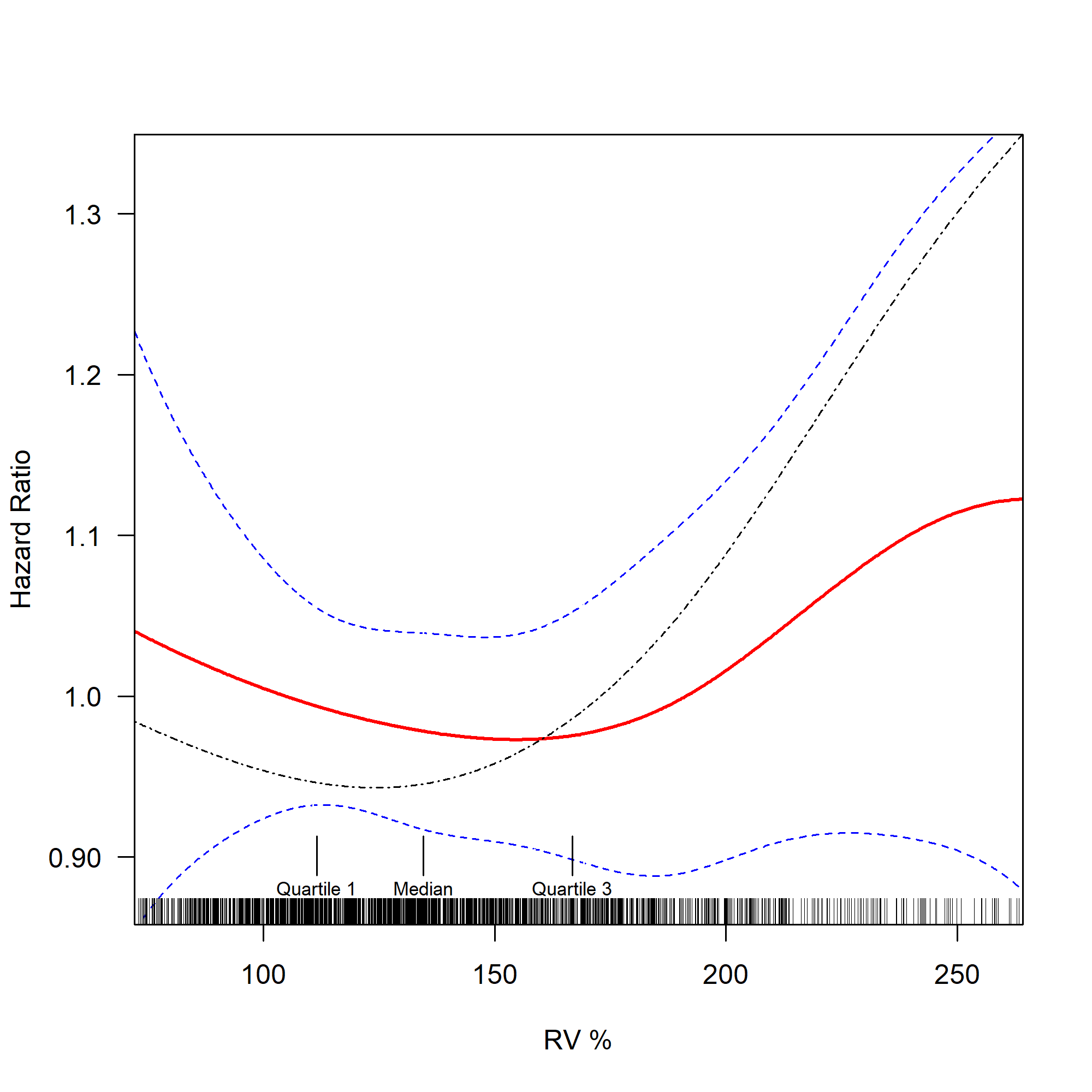 |
| --- | --- |
| 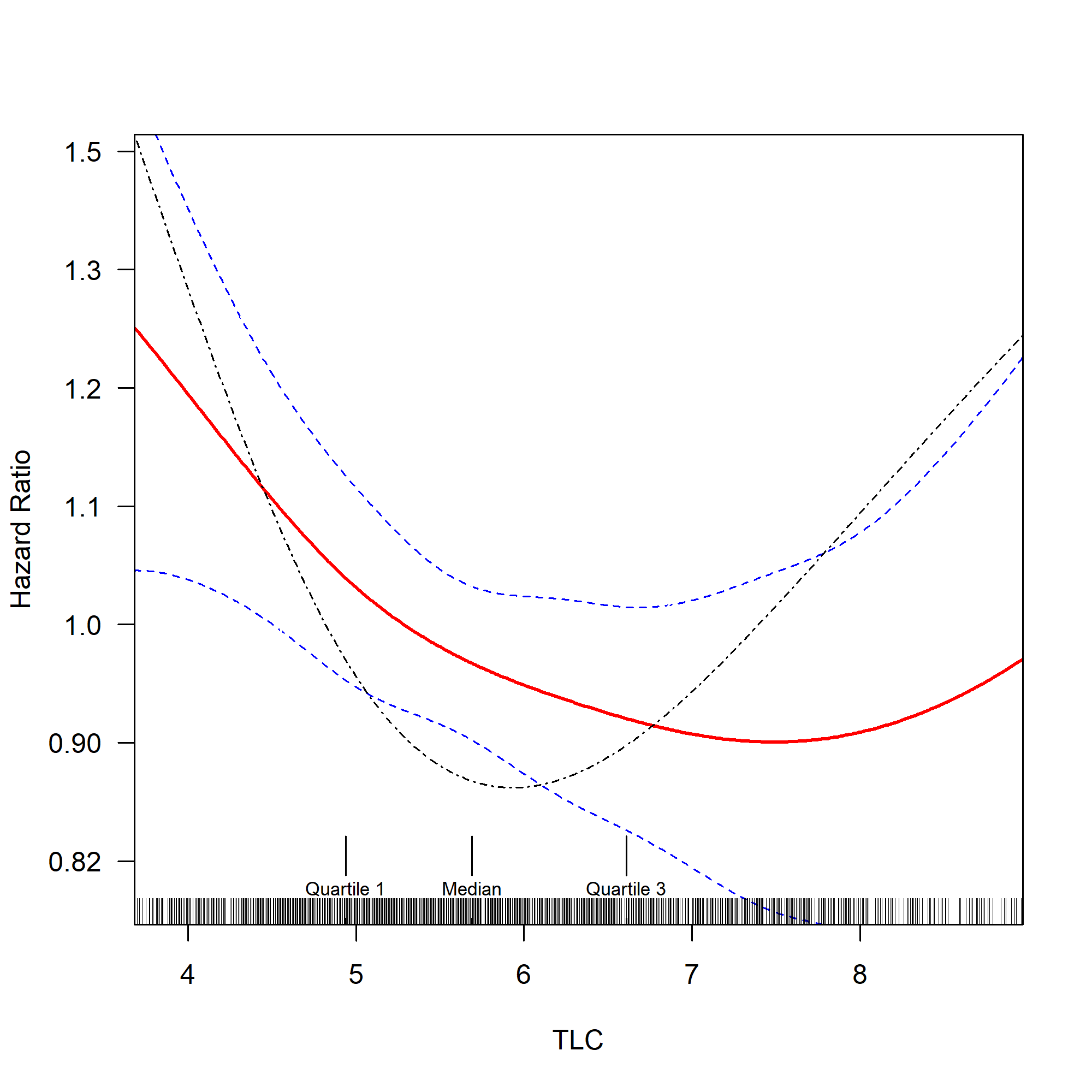 | 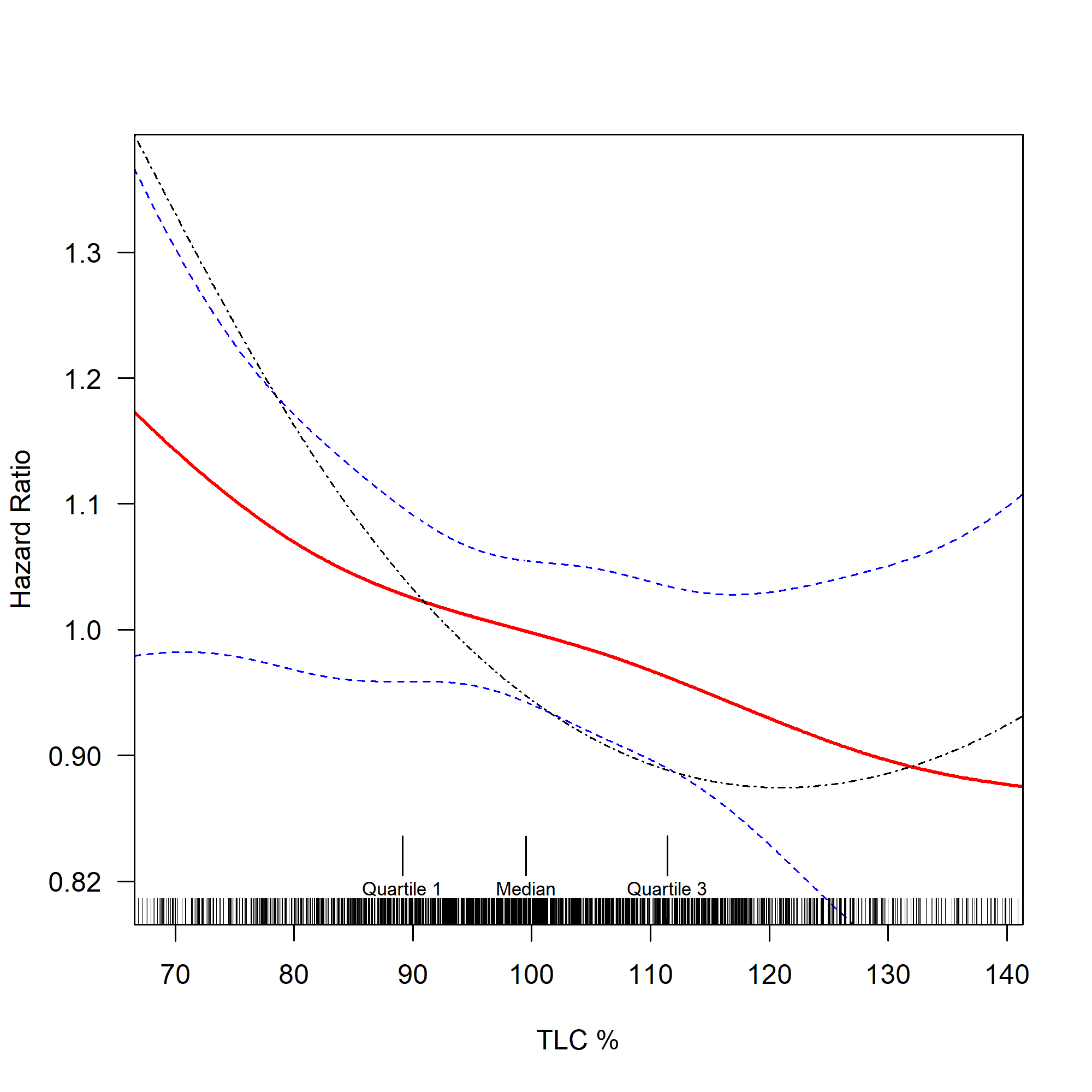 |
| 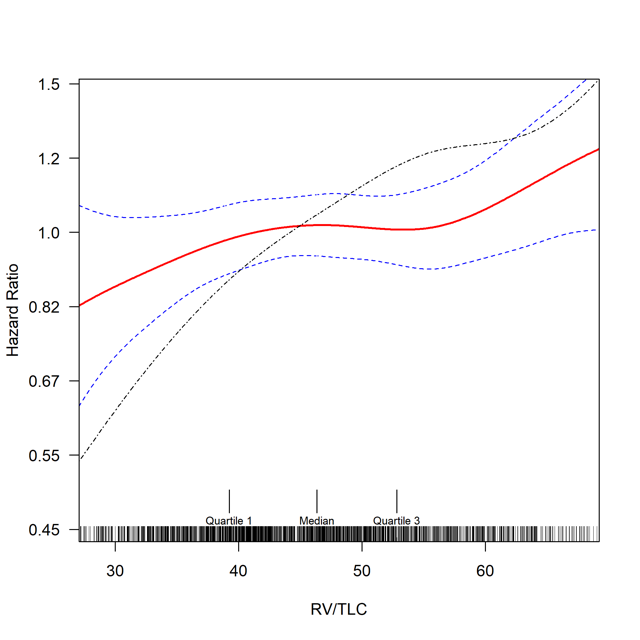 | 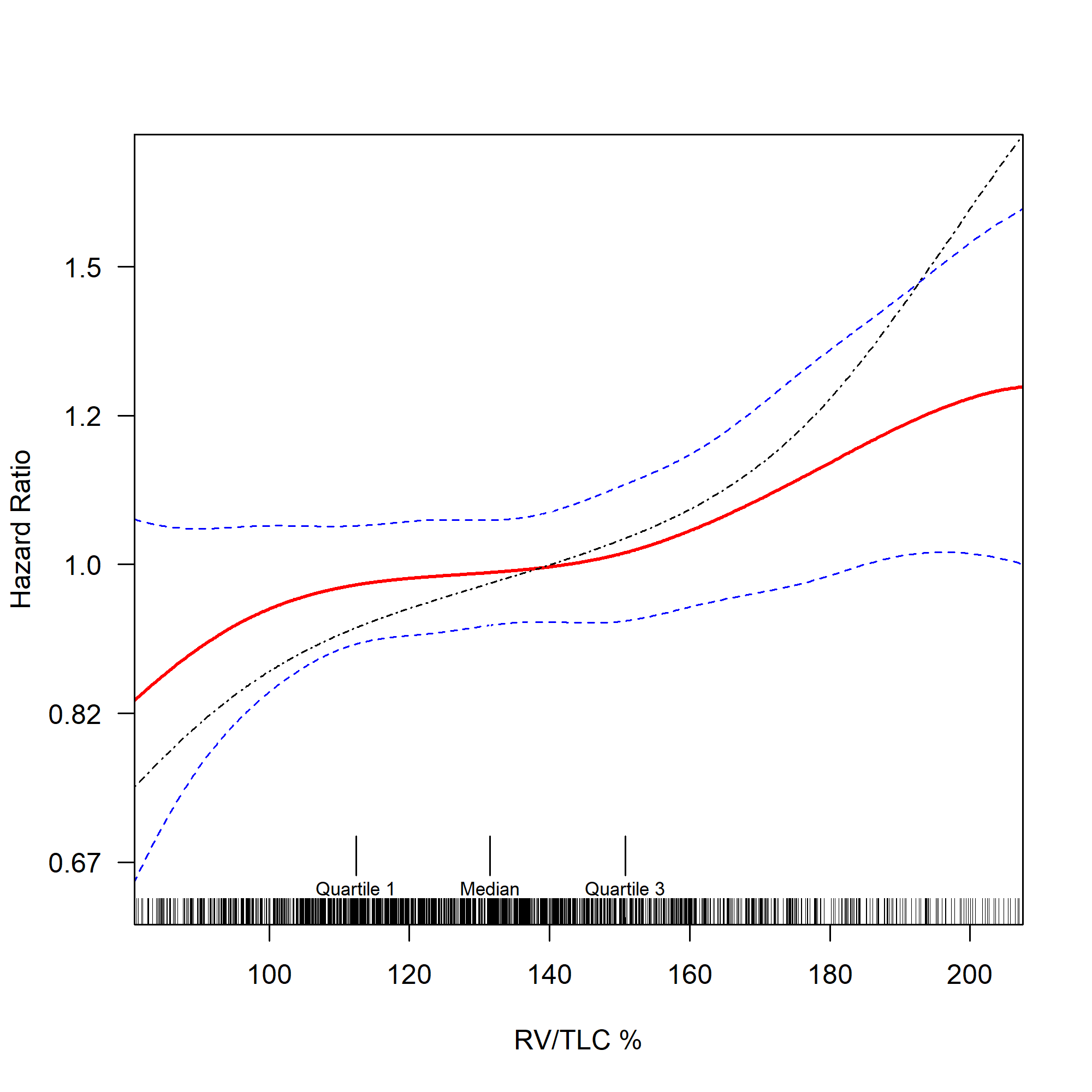 |
| **Figure S3. Shape of associations between lung volumes and risk of death in NSCLC patients.** Unadjusted (black dot-dashed lines) and adjusted hazard ratios (red solid line) with 95% confidence intervals (blue dashed lines) were obtained from spline Cox regression model of overall survival with each lung volume test as a continuous predictor. The X axis ranges from the 5^th^ to 95^th^ percentile of each test. Abbreviations: RV = residual volume; TLC = total lung capacity. | |

**
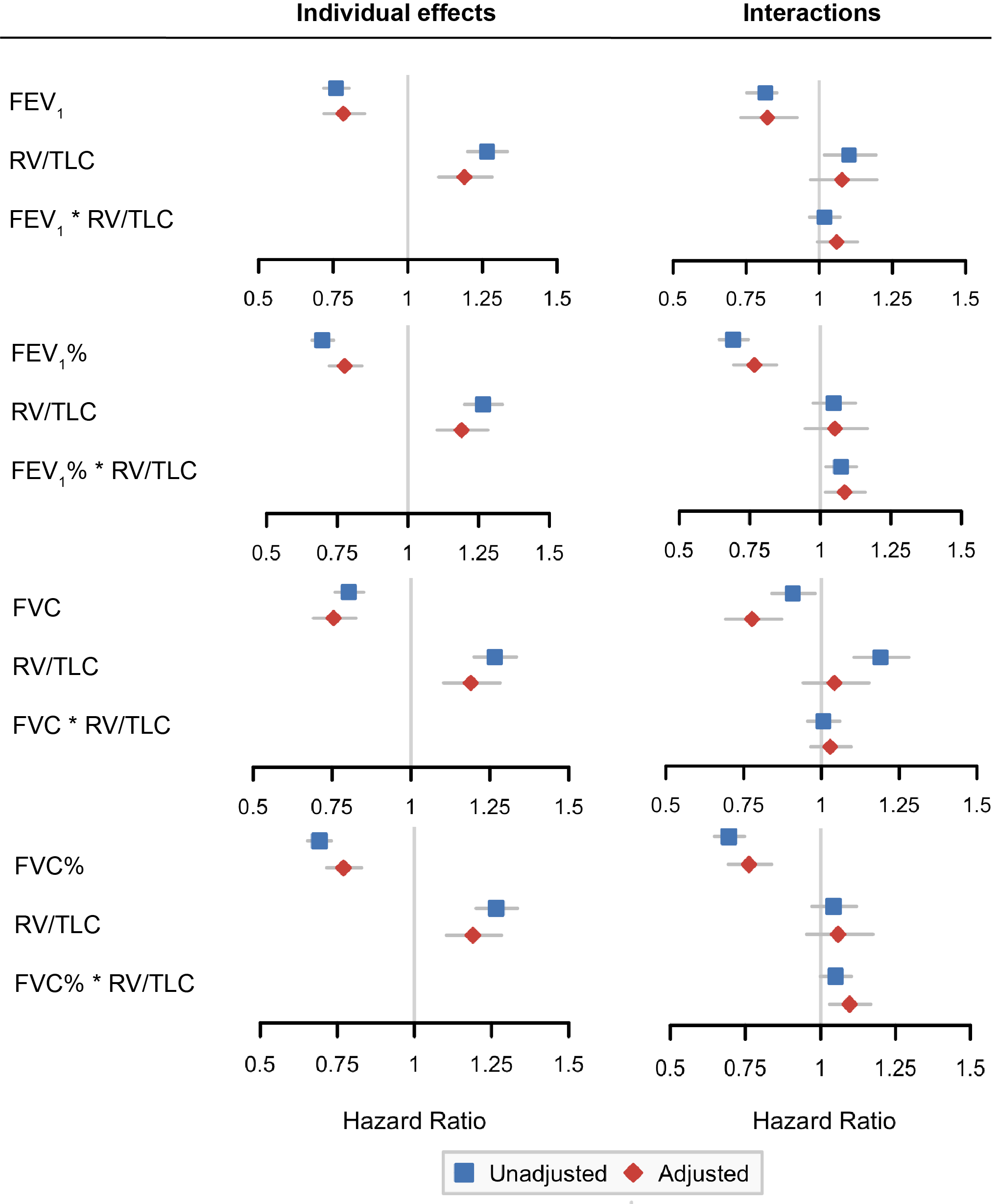
**

**Figure S4. RV/TLC versus spirometry: individual effects and interactions on risk of death in NSCLC patients.** The hazard ratios were reported as relative changes per 1 standard deviation increment in the corresponding covariates. Individual effects were measured without adjusting for another variable of interest or their interactions; interactions were reported as hazard ratios of each term of interest in the interaction model. Adjusted hazard ratio denotes the association accounting for age, sex, BMI, smoking, NSCLC histological subtypes, clinical stages, lung cancer treatments and their time varying effects. Abbreviations: NSCLC = non-small cell lung cancer; FEV_1_ = forced expiratory volume in 1 second; FVC = forced vital capacity; RV = residual volume; TLC = total lung capacity.

| **A**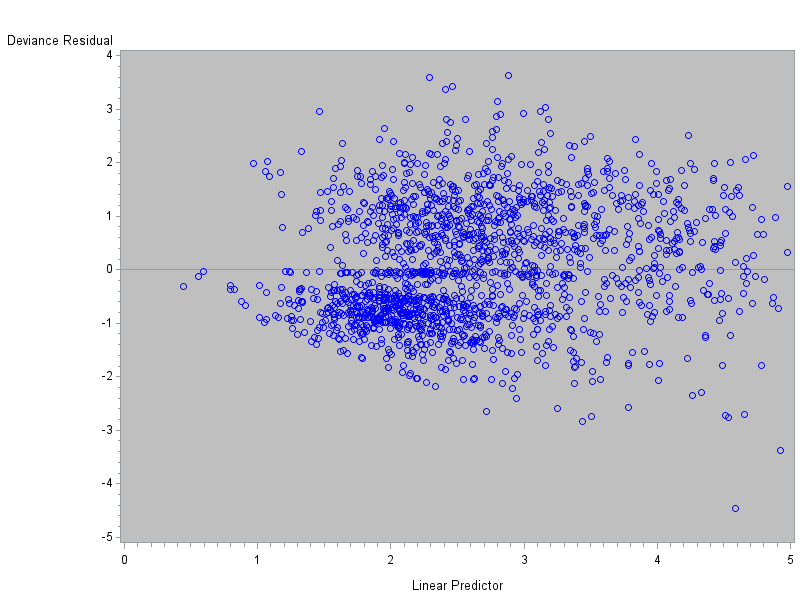 | **B**  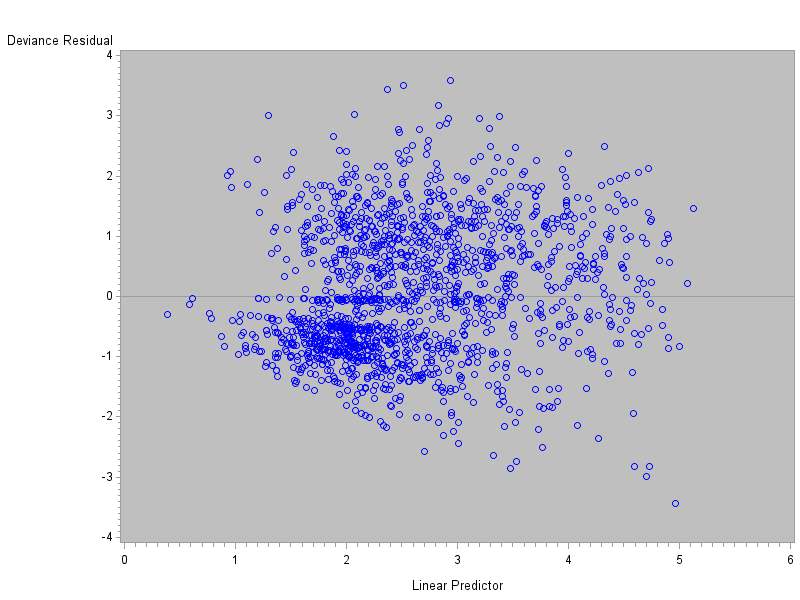 |
| --- | --- |
| **C**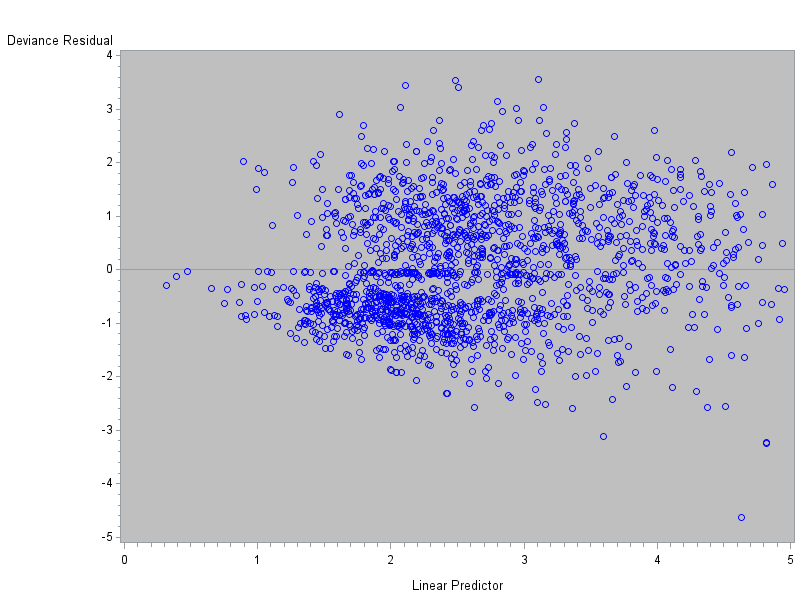 | **D**  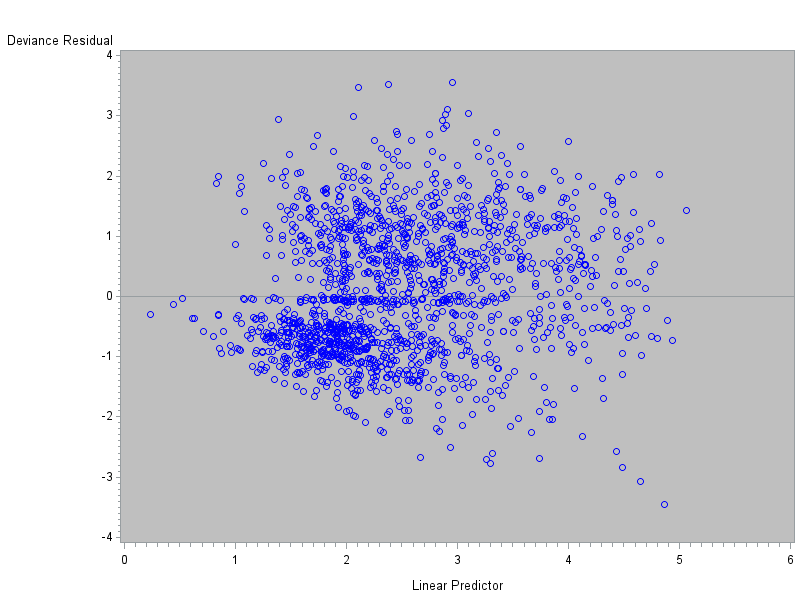 |
| **E**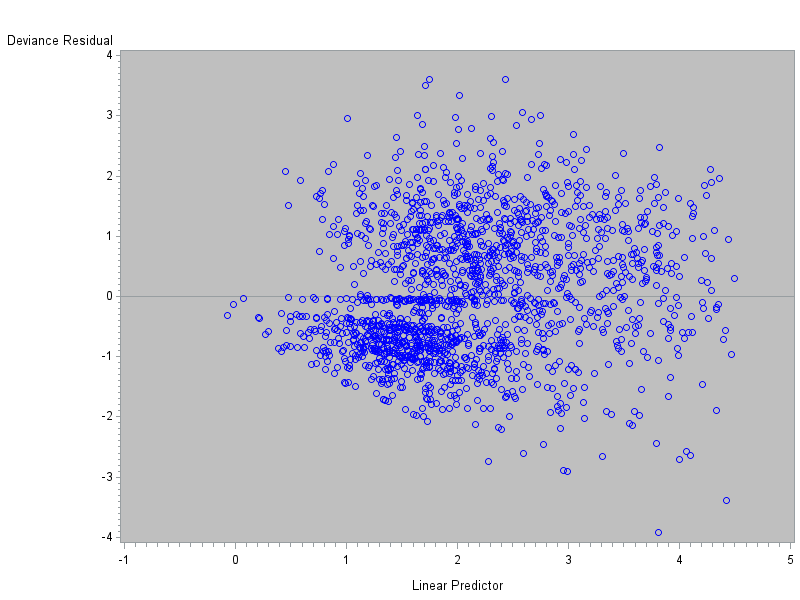 | **F**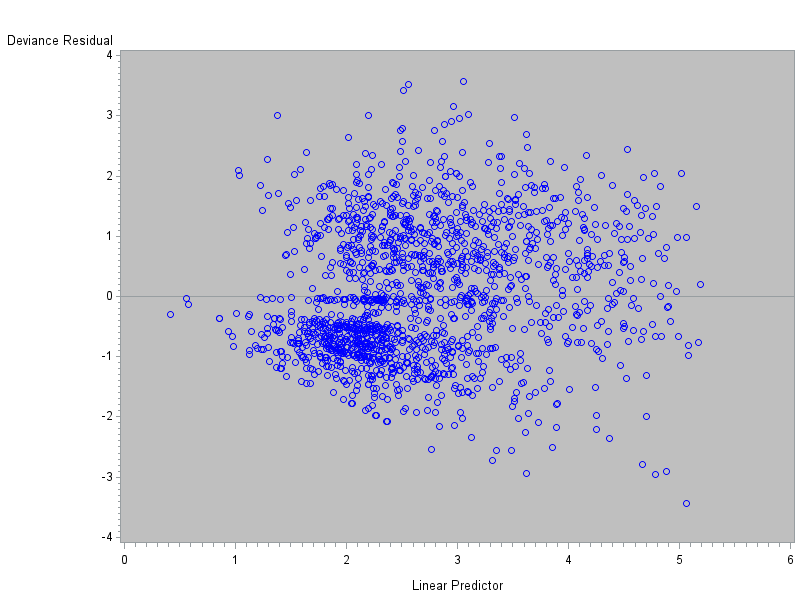 |
| **G**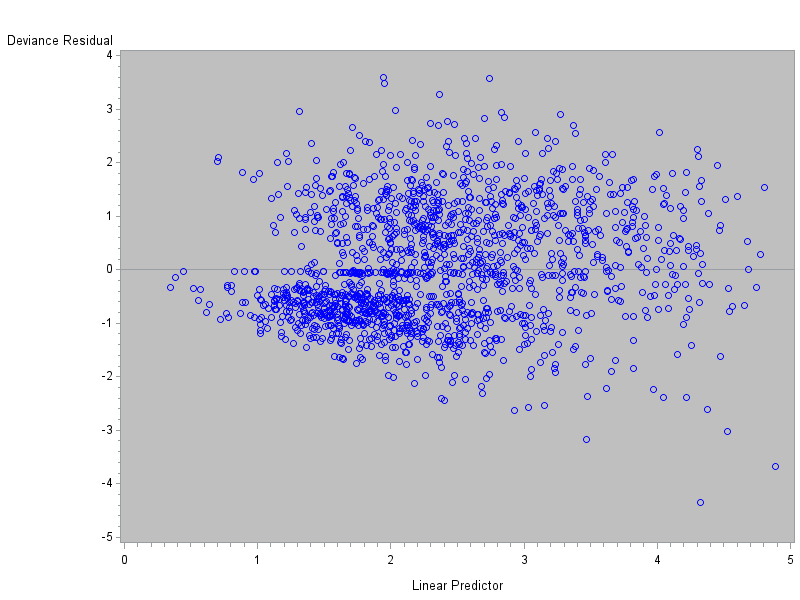 | **H**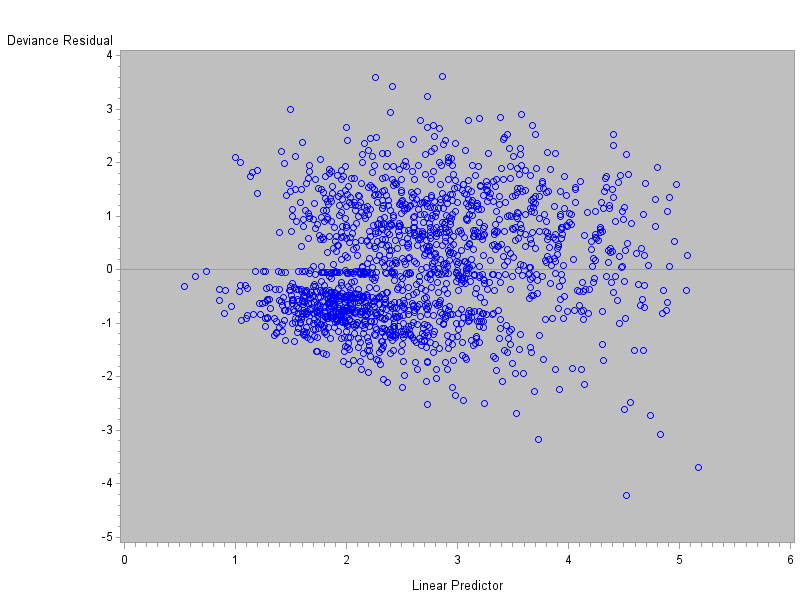 |

**Figure S5. Deviance plots for Cox models in the main analyses.** RV (A), RV% (B), TLC (C), TLC% (D), RV/TLC (E), RV/TLC% (F), DLCO (G), DLCO% (H) were modeled with adjustments for age, sex, BMI, smoking, histology, stage, and stratified on treatment.
